# Recent trends in the stereoselective synthesis of (poly)-substituted 2-oxo acids by biocatalyzed aldol reaction

**DOI:** 10.1016/j.cogsc.2021.100476

**Published:** 2021-03-10

**Authors:** Mathias Pickl

**Affiliations:** Institute of Chemistry, University of Graz, Heinrichstrasse 28, 8010, Graz, Austria

## Abstract

Recently, an increased interest toward enzymatic carboligation was observed, as biocatalytic carbon–carbon bond formation is a common obstacle in retrosynthetic planning. The construction of extended 2-oxoacid frameworks by 2-oxoacid aldolases and enzymes acting as aldolases is a potent tool for synthetic chemists since a broad spectrum of downstream reactions through functional group interconversions gives access to a plethora of compound classes. In the search for selective biocatalysts, successful protein engineering efforts and high throughput screenings from biodiversity expand the structural diversity of nucleophile and electrophile substrates. Several successful examples with an emphasis on reactions catalyzed by class II aldolases and enzymes acting as class II aldolases are highlighted, including reactions in which both enantiomeric products and in selected cases even diastereomeric products are accessed.

## Introduction

As biocatalysis is progressively implemented in pharmaceutical processes it is notable that many functional group interconversions are feasible; however, the demand in selective carbon—carbon forming reactions remains underexploited [1–5]. The biocatalytic alternatives to conventional synthetic routes contribute to the sustainability of the overall chemical process since the parameters of enzymatic reactions align predominantly with the principles of green chemistry especially regarding chemoselectivity, regioselectivity, and stereoselectivity [6,7]. Nonetheless, the construction of a complex organic framework starting from simple achiral building blocks is a challenge with available biocatalytic tools. The current limitations are found in the stereoselectivity in the carboligation reaction and the lack of complementary catalysts in case selective enzymes were identified. Furthermore, the substrate scope is considered rather narrow, which reduces drastically the potential applications. Fortunately, recent efforts brought significant improvements in selectivity and performance of biocatalysis in selective carbon—carbon bond formation [8–10]. A class of lyases labeled as aldolases is recently getting rediscovered since it reliably performs the highly desired biocatalytic carbon—carbon bond formation [11]. Particularly, pyruvic acid aldolases deserve more attention in view of their potential for the biocatalytic community. The modularity of the aldol reaction catalyzed by pyruvic acid aldolases elongating a broad pool of aldehydes with 2-oxoacids into 4-hydroxy-2-oxoacid products is impressive. The constructed carbon frameworks with this approach are highly valuable due to the manifoldness of the established downstream routes such as 2,4-dihydroxy acids, 2-amino-4-dihydroxy acids, or 3-hydroxy acids [12]. In the last few years, the attractiveness of aldolase-mediated carbon—carbon bond formation has risen due to the progress in enzyme discoverynd protein engineering efforts. Several obstacles such as the narrow substrate scope and a lack of stereoselectivity of this reaction have been targeted and the progress will be highlighted in this review.

**Scheme 1:**
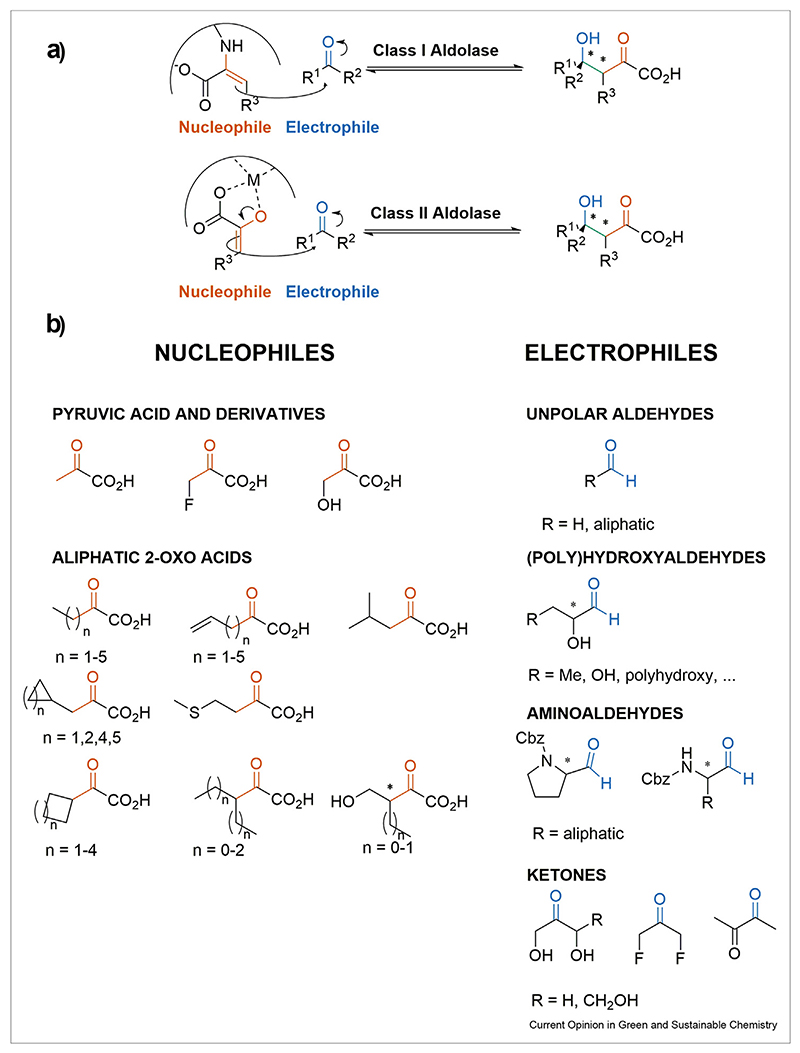
Aldolase-mediated carboligation with the current scope of nucleophiles and electrophiles. (**a**) Aldolase-mediated aldol reaction furnishing elongated 2-oxo acid frameworks. A carbonyl compound as an electrophile (Blue) and a 2-oxo acid as a nucleophile (Orange) form a carbon-carbon bond (green). The mechanism of class I and class II aldolases are depicted: The Mechanism of class I aldolases bearing an active site lysine residue and below class II aldolases in which an active site divalent metal ion binds to an enolized pyruvic acid that attacks the carbonyl of an aldehyde. (**b**) The expanded nucleophile scope and electrophiles for the aldol reaction of 4-hydroxy-2-oxo acids mediated by pyruvic acid aldolases.

**Scheme 2:**
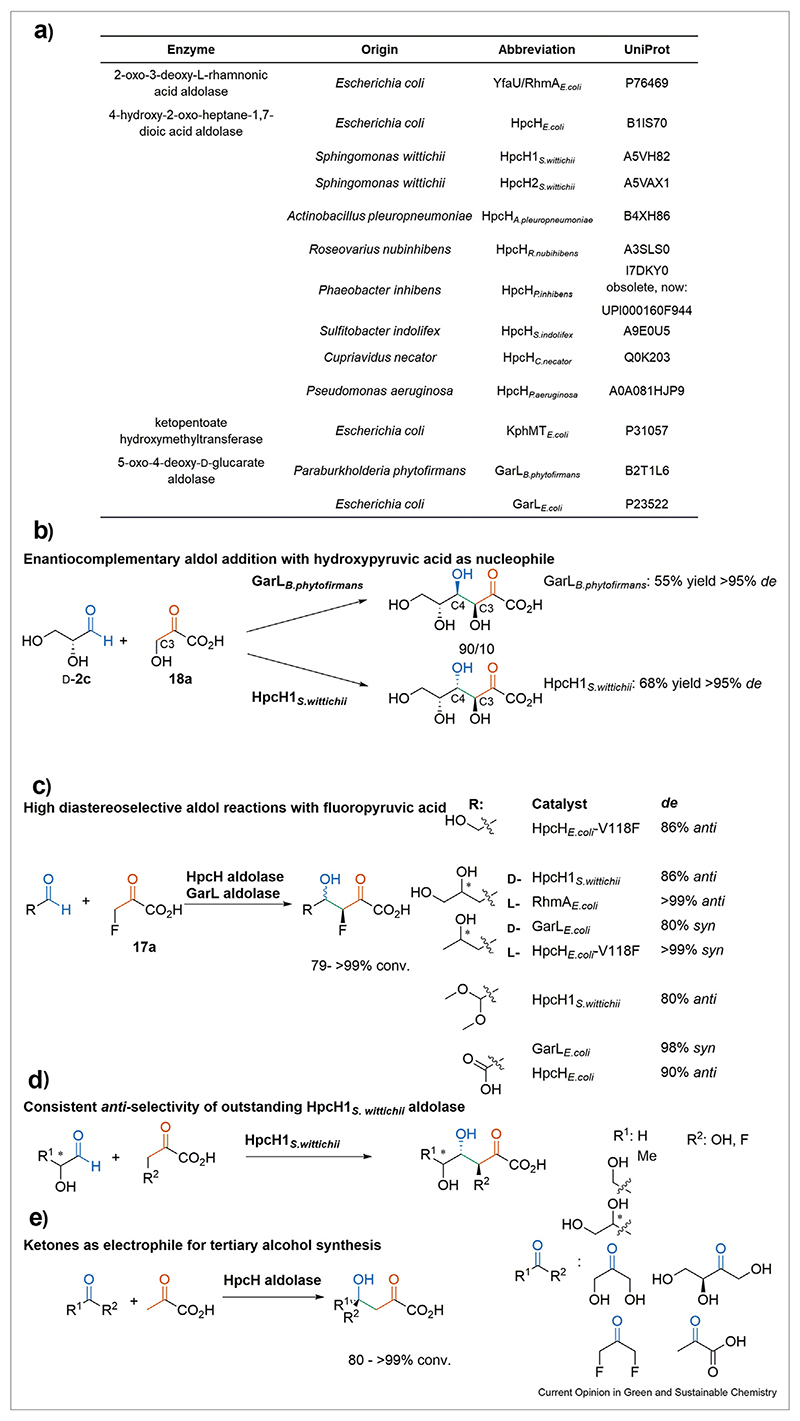
An overview of potent class II aldolases and enzymes acting as class II aldolases and examples of stereoselective aldol additions for the synthesis of (poly)substituted 2-oxo acids. (**a**) A list of class II aldolases and enzymes acting as aldolases discussed in this review, including their source, UniProt code, an abbreviation used in this review. (**b**) Hydroxypyruvic acid as a nucleophile in an aldol reaction with d-glyceraldehyde by 4-hydroxy-2-oxo-heptane-1,7-dioic acid aldolase (HpcH aldolase) or 5-oxo-4-deoxy-d-glucarate aldolase (GarL aldolase) from various organisms [22]. (**c**) Fluoropyruvic acid as a nucleophile in class II aldolase-mediated reactions [29]. (**d**) 2-hydroxyaldehydes as substrates for HpcH1_*S.wittichii*_ aldolase showing antiselectivity with fluoropyruvic acid and hydroxypyruvic acid. (**e**) Ketones as an electrophile for the creation of tertiary alcohols [23].

A pyruvic acid, or more generally, 2-oxoacid aldolase-mediated reaction forges a carbon—carbon bond between a 2-oxoacid, acting as a nucleophile in its enolized form and a second carbonyl acting as an electrophile (Scheme 1a). This furnishes an aldol adduct with up to two stereocenters with optimal atom efficiency. To be in a reactive state, the nucleophile referred to as ‘aldol donor’ undergoes enolization promoted by an active site base [13]. Hence, the active site of aldolases is designed based on the ability to shield this highly reactive intermediate from the bulk solution. Consequently, the nucleophile binding site of the enzyme has a rather stringent substrate selectivity tolerating only a few structural alterations because of its high steric and electronic constraints. These constraints are required to maintain a highly ordered bi-substrate transition state that occurs upon the admission of the electrophile i.e., ‘aldol acceptor’ to assure a perfect stereochemical outcome. The most established aldolases are the so-called ‘class I aldolases’ with an active site lysine to form a Schiff base with the nucleophile (Scheme 1a) [14]. Remarkably, the emerging protocols of aldolase-mediated reactions rely almost exclusively on the hitherto underappreciated metal-dependent class II aldolase family. In these enzymes, the activation of the nucleophile proceeds via enolization driven by a divalent Lewis acidic metal ion located in the active site of the enzyme (Scheme 1a). The range of metals in class II aldolases are found among a variety of transition metals such as nickel, cobalt, manganese, or zinc but also alkaline earth metal magnesium [15]. In their sequence, usually, one characteristic protein domain is found that allows the assignment of the aldolases beyond the catalytic species. The most promising class II aldolases belong to the same Pfam family [PF03328] named 4-hydroxy-2-oxo-heptane-1,7-dioic acid (HpcH)/HpaI aldolase/citrate lyase family (Scheme 2a). The manifoldness in the genomic context of this family suggests a diverse range of physiological activities. HpcH aldolases, however, have been identified to either participate in the last steps of the degradation of biphenyl or polychlorinated biphenyl in a retro-aldol manner or as the related 5-oxo-4-deoxy-d-glucarate aldolases (GarL) in the catabolism of d-glucarate/galactarate [16–18].

Enzymes of the [PF03328] mechanistic type adopt the common TIM barrel fold and are hexamers (i.e., trimer of dimers) binding one metal cofactor per subunit. The metal itself binds by an aspartate and a glutamate over a water bridge. An arginine residue in the active site is responsible for the binding of the nucleophile carboxylate and together with an adjacent histidine are identified to be crucial for enzymatic activity, participating in the activation of metal-bound water for proton transfer during catalysis [19,20]. Hence, mostly small nucleophiles as pyruvic acid and its analogs were explored due to the restrictive architecture of the active site.

## Recent findings with pyruvic acid and 3-substituted analogs as nucleophiles

Pyruvic acid is not only eponymous for pyruvic acid aldolases, but also the best-investigated nucleophile [21]. Fortunately, class II aldolases accept its analogs, hydroxypyruvic acid and fluoropyruvic acid, which enables the scope of accessible products. The most successful electrophiles within these studies are small chiral (poly)hydroxylated aliphatic aldehydes such as glycolaldehyde, glyceraldehyde, lactaldehyde or erythrose. A notable observation is that enzymes of the HpcH/HpaI aldolase/citrate lyase family accept both the *S*-configuration and the *R*-configuration at the 2-hydroxy group in the electrophilic aldehyde. Furthermore, although both *syn*- and *anti*-diastereoselectivities are described, the configuration of the chiral center at C3 is exclusively *S*-oriented [22,23].

### Hydroxypyruvic acid as nucleophile

In case hydroxypyruvic acid is the selected nucleophile, the hydroxy substituent on the C3-position enables the construction of a 3,4-dihydroxy-2-oxoacid motif with the control over both stereocenters (Scheme 2b), which is a long-lasting goal within aldolase research [22]. Due to its self-activating capacity, the spontaneous homo-aldol addition of hydroxypyruvic acid is an obstacle, which must be overcome [23]. In a screening of 571 class II aldolases, 19 wild type enzymes of the HpcH/HpaI/citrate lyase family, tolerating hydroxypyruvic acid as a nucleophile, were found [22]. The remarkably selective enzyme HpcH1_*S.wittichii*_ (Scheme 2b and 2d), which catalyzes the aldol addition of hydroxypyruvic acid to enantiopure 2—hydroxyaldehydes yielding aldol, adducts with a remarkably high diasteroselectivity and isolated yields ranging from 47% to 68% with the products showing solely *S*,*S*-configuration. Moreover, d- or l-glyceraldehyde have given 68% and 61% isolated yields, respectively, l-lactaldehyde (47% isol. yield) and d-erythrose (57% isol. yield, >95% *de*) the excellent *de* of the *anti*-3,4-hydroxy-2—oxoacid product highlights the great potential of the catalyst. HpcH1_*S.wittichii*_ lacks only d-lactaldehyde in this distinct selectivity with 55% *de* [22]. The conversion rate of hydroxypyruvic acid is relying on an active site phenylalanine side chain in HpcH1_*S.wittichii*_ in which a CH-π interactions to the C3 of the enolized nucleophile occurred. Replacing the phenylalanine residue with other aromatic amino acids such as tyrosine offers a 2-fold improvement [24]. High selectivity is also found with GarL_*B.phytofirmans*_ with noteworthy enantiocomplementary 3*S*,*4R*-configuration obtained with similar yield (Scheme 2b, 55%) [22].

### Fluoropyruvic acid as nucleophile

Fluorine substituents provide unique options to new organofluorine core structures, offering great pharmacological potential [25,26]. The groundbreaking work of Berry and co-workers with fluoropyruvic acid as nucleophile utilizes class I aldolases, thus, not the scope of this review and is reviewed here in detail [26–28]. With class II aldolase HpcH_*E.coli*_ a change of the nucleophile to fluoropyruvic acid over pyruvic acid resulted in a 500-fold decrease in the turnover number and was accompanied by substrate inhibition. This may be overcome in a continuous substrate addition process [29].

A broad scope of aldehydes is suitable as electrophiles for fluoropyruvic acid in an aldol addition mediated by members of the HpcH/HpaI/citrate lyase family (Scheme 2c) [29]. A screening found high acceptance toward 2-hydroxyaldehydes, aliphatic aldehydes, 2,2-dimethoxyacetaldehyde, and even glyoxylic acid. Exclusively the 3*S*-configuration is present in the products; however, the configuration and enantiopurity of the second chiral center is largely dependent on the electrophile, e.g., the *S*-configuration in l-lactaldehyde leads to a strong preference toward the *anti*-isomer regardless of the employed HpcH aldolase (HpcH_*E.coli*_: 99% conv. 92% *de*, HpcH1_*S.wittichii*_ 100% conv. 90% *de*, HpcH2*_s.wittichii_* 87% conv. >99% *de*). In case d-lactaldehyde is employed GarL_*E.coli*_ furnishes the *syn*-product in a complementary fashion (97% conv., 80% *de*). The only exception remains d-lactaldehyde with 1:1 ratio of *syn/anti* product. An observation that is also made in case d-lactaldehyde reacts with hydroxypyruvic acid as nucleophile [22]. Furthermore, the *anti*-product (up to >99% *de*) is obtained in case l-glyceraldehyde is employed with the HpcH aldolases and GarL*_E.coli_* with up to 97% conversion [29].

The *de* may be reduced over time toward the thermodynamic product and therefore requires kinetic control [27,30]. Lowering the catalyst loading boosts the *anti*-selectivity with the electrophiles glycoaldehyde and 2,2-dimethoxyacetaldehyde from 39–59% to 71–93% *de*-values *anti*, which is accompanied by a drop in conversion [29]. Surprisingly, in the case of glyoxylic acid as electrophile GarL_*E.coli*_ turned out as a potent catalyst with extraordinary diastereomeric selectivity toward *syn* (99% *de*) and full conversion whereas HpcH_*E.coli*_ produced the *anti*-product (92% *de*) [29].

## Ketones as alternative electrophiles for pyruvic acid analogs

The pyruvic acid self-addition has revealed the feasibility of ketones acting as electrophiles for an aldol reaction. The produced tertiary alcohols are a widespread motif in bioactive compounds [31]. Since conventional organic synthesis uses hazardous metals and chemicals biocatalytic alternatives were developed such as the use of thiamine-dependent lyases and hydroxynitrile lyases to furnish 2-alkyl-2-hydroxy ketones, 2-alkyl-2-hydroxy-3-diketones, 3-hydroxy ketones, and ketone cyanohydrins [32]. Recent successful efforts used dihydroxyacetone phosphate-dependent class II aldolases furnished tertiary alcohol moieties in branched-chain sugars in an enantioselective fashion [33]. Hence, the feasibility of HpcH aldolase for the synthesis of tertiary alcohols bearing a carboxy group has been probed with hydroxyacetone and analogs thereof. It turned out that a single hydroxyl group adjacent to the ketone is not sufficient that hydroxyacetone is accepted as an electrophile; however, dihydroxyketone is readily accepted by a broad set of HpcH aldolases (Scheme 2e) [23]. The most promising enzyme candidates are HpcH2_*S.wittichii*_, HpcH_*A.pleuropneumoniae*_, HpcH_*S.indolifex*_, and HpcH*_R.nubihibens_* with pyruvic acid and 2-oxobutyric acid as nucleophiles. Besides dihydroxyketone, its fluoro-analog, 2,3-butadione, and keto sugar l-erythrulose are also accepted. The best catalyst performs aldol addition onto the ketones at similar rates as with aldehydes; however, it was required to provide the ketones in a fourfold excess to shift the equilibrium toward product formation. Yields are up to 75% with dihydroxyacetone and 90% with l-erythrulose and 2-oxobutyric acid by using HpcH2_*S.wittichii*_ as a catalyst. Pyruvic acid as nucleophile has a slight edge since with dihydroxyacetone as an electrophile and 92% of the aldol product were isolated. The products are found to be in *S*-configuration at the chiral center at C4 [23].

## The extension of the aliphatic chain length of the nucleophilic 2-oxoacids

**Scheme 3:**
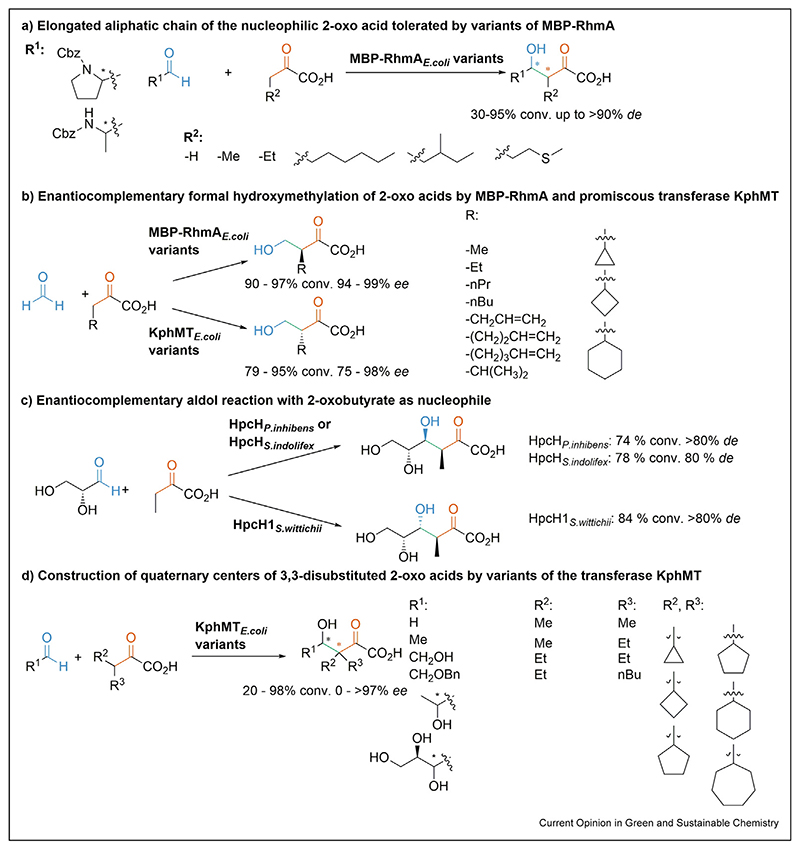
The expanded nucleophilic scope of class II aldolases. (**a**) Protected aminoaldehydes as electrophiles for class II aldolase MBP-2-oxo-3-deoxy-l-rhamnonate aldolase (RhmA*_E.coli_*) and its variants [35]. (**b**) Enantiocomplementary aldol addition of formaldehyde with MBP-RhmA_*E.coli*_ and oxopentoate methyltransferase (KphMT_*E.coli*_) variants [38]. (**c**) Stereocomplementary aldol addition with 2-oxobutyric acid as a nucleophile and exemplarily with d-glyceraldehyde as electrophile mediated by HpcH aldolases [39]. (**d**) Recently identified KphMT*_E.coli_* variants are probed with 3,3-disubstituted 2-oxo acids and tolerate large (poly)hydroxylated aldehydes to form quaternary centers with low to high enantioselectivity [40].

The limitation of the size of the nucleophile is one of the key obstacles working with 2-oxo acid aldolases. Hence, one focus point of recent investigation was the enlargement of the aliphatic chain of the employed 2-oxo acids. Structure-guided protein engineering with the purpose of active site enlargement of class II aldolase 2-oxo-3-deoxy-l-rhamnonate aldolase (RhmA_*E.coli*_) also annotated as YfaU, which was fused to the maltose-binding protein (MBP) to improve soluble expression [34]. The engineering efforts ultimately enabled a broad scope of aliphatic 2-oxo acids as nucleophiles such as 2-oxobutyric acid, 2-oxopentanoic acid, 2-oxooctanoic acid, oxoleucine, and oxomethionine (Scheme 3a). Bulky residues within the active site were replaced and the resulting variants MBP-RhmA_*E.coli*_-W23V, MBP-RhmA_*E.coli*_-L216A, and MBP-RhmA_*E.coli*_-W23V/L216A were probed with both enantiomers of Cbz-protected prolinal and alaninal that are attractive electrophiles as the obtained aldol products are precursors for proline, pipecolic acid, and even more widespread 4-amino-3-hydroxy acid motif [35,36]. Conversion ranged from 30 to 95%, while both enantiomers of the electrophiles were accepted. The chiral center in the aldol product is preferentially *S*-configured at the 3-position with *des* up to >90% [35]. Best diastereoselectivity is founh oxomethionine (91% conv.), or 2-oxooctanoic acid (40% conv.) and (*R*)-Cbz-alaninal (*syn*, selectivity of >90% *de*). An apparent change in the active site orientation in case oxoleucine acted as a nucleophile (50% conv.) lead to a switch in the diastereomeric outcome (*anti* 90% *de*) with (*S*)-Cbz-alaninal.

A similar engineering attempt expanded the active site of promiscuous tetrahydrofolate dependent oxopantoate hydroxymethyltransferase (KphMT_*E.coli*_) of the Pfam family [PF02548]. In a co-factor independent mechanism KphMT_*E.coli*_ utilizes a Lewis-acidic divalent transition metal such as cobalt to act as a class II aldolase [37]. With the variants KphMT*_E.coli_*-I202A and KphMT_*E.coli*_-I212A a similar nucleophile scope as that of MBP-RhmA_*E.coli*_ is observed. The synthetic potential of these variants lies in the stereocomplementary behavior of MBP-RhmA_*E.coli*_, which was revealed using formaldehyde as an electrophile (Scheme 3b). Furthermore, similarly to MBP-RhmA_*E.coli*_ also KphMT_*E.coli*_ variants tolerate 3-cyclopropyl-2-oxoacid and 3-cyclobutyl-2-oxoacid whereas 3-cyclohexyl-2-oxoacid is exclusively tolerated by MBP-RhmA_*E.coli*_ [38].

An NMR-based screening assay revealed that 2-oxobutyric acid is well tolerated by several wild-type HpcH aldolases [39]. 2-oxobutyric acid was tested on *R*-configured 2-hydroxyaldehyde electrophiles. In case d-glyceraldehyde was employed, both *syn*-diastereomers and *anti*-diastereomers are accessible with up to >84% product formation and >80% *de* using HpcH1_*S.wittichii*_ and HpcH_*P.inhibens*_ (Scheme 3c). The *anti*-selectivity correlates with the other 3-substituted nucleophiles when HpcH1_*S.wittichii*_ is employed (d-glyceraldehyde 70% *de*, d-lactaldehyde 50% *de*, d-erythrose 80% *de*). The bulky d-ribose leads to a significant drop in selectivity accompanied by a switch in diastereoselectivity (*syn* 20% *de*). HpcH_*S.indolifex*_ with a remarkably high *syn*-preference [39] has the potential to become a working horse for biocatalytic aldol reactions since its consistent selectivity found with the tested nucleophiles is highly desired.

KphMT_*E.coli*_ wild type and variants also are suitable for the unprecedented construction of quaternary carbon centers [32,40]. It turns out that ramifications at the 3-positions within the nucleophile present already in the natural oxopantoate pathway were also tolerated in case KphMT_*E.coli*_ acts in an aldolase fashion. This activity allows the construction of 3,3,3-trisubstituted 2-oxoacids with *gem*-alkyl and spirocyclic centers (Scheme 3d). Besides small aldehydes such as formaldehyde, acetaldehyde, and *R*- or *S*-lactaldehyde, C4-aldosugars and C5-aldosugars are tolerated as electrophiles. Bulky cyclopentyl- (90% conv.), cyclohexyl- (20% conv.), and cycloheptyl-2-oxoacetic acid (40% conv.) are tolerated as nucleophile onto formaldehyde in an analytical scale screening and the aldol products are directly decarboxylated and esterified on a preparative scale. Small 3,3-disubstituted 2-oxo acids provide reasonable stereoselectivity with up to >80% *ee* with formaldehyde as an electrophile. The most active variants KphMT_*E.coli*_-I202A and KphMT_*E.coli*_-I212A and the corresponding double mutant provide more than 90% conversion. The variant KphMT_*E.coli*_-V214G is particularly potent for the ligation of 2-oxoisovaleric acid and d-erythrose. Furthermore, it turns out that KphMT_*E.coli*_ variants enantioselectively convert only the *S*-enantiomer of Ketoisoleucine. Molecular models suggest that the steric repulsion of active site residues limits the accessibility of the enolate intermediate [40].

## Follow-up chemistry for 4-hydroxy-2-oxo acids produced by an aldol reaction

**Scheme 4:**
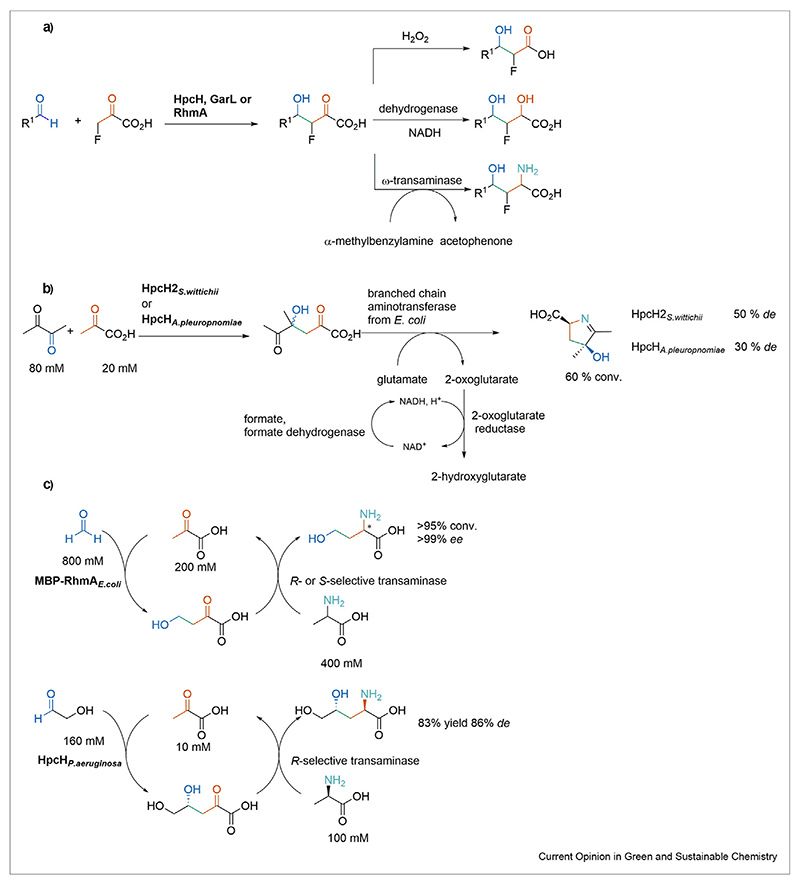
Biocatalytic follow-up chemistry for 2-oxo acids furnished by aldol addition. (**a**) One-pot two-step follow-up sequence for fluoro-aldol products [29]. (**b**) One-pot simultaneous cascade of an aldol reaction and a subsequent trans-amination [23]. (**c**) One-pot cyclic cascade of an MBP-RhmA*_E.coli_* mediated aldol addition and transamination [34] and HpcH*_P.aeruginosa_* and trans-amination [45].

The versatility of 2-oxoacid intermediates in biocatalytic multistep routes or as a platform for broad product portfolios is well described [41–43]. The scarcity of one-pot one-step reactions for downstream modifications of aldol products can be explained by the reversibility of the aldol reaction [23,34,44]. Therefore, inactivation of the aldolase by precipitation or removal of the active site metal ion is often required for further follow-up chemistry [29]. This methodology enables the reduction of the 2-oxo group of the product by dehydrogenase and the amination by a u-transaminase (Scheme 4a).

A remarkable example is a one-pot one-step cascade couples an aldol reaction of pyruvic acid and butanedione mediated by HpcH2_*S.wittichii*_ and HpcH_*A.pleuropneumoniae*_ with the amination of a branched-chain aminotransferase from *Escherichia coli* (Scheme 4b). The equilibrium shift is driven by the removal of the deaminated amino donor by 2-oxoglutarate reductase coupled with a cofactor recycling system. A one-pot cyclic reactions setup starting from pyruvic acid, formaldehyde, and alanine as amino source provided an efficient route for the synthesis of both enantiomers of homoserine. MBP-RhmA*_E.coli_* and enantiocomplementary set of ω-transaminase tolerated 400 mM substrate loading and produced homoserine between 86% and >95%, respectively, with >99% *ee* for both enantiomers (Scheme 4c). A related approach synthesized l-*syn* or *anti*-4-hydroxyglutamic acid and d-*anti*-4,5-dihydroxynorvaline with 83–95% yield. In the case of d-*anti*-4,5-dihydroxynorvaline extraordinary 86% *de* was achieved in a preparative scale experiment (Scheme 4c) [45]. Cornerstones for the experimental setup proved to be the slow addition of the electrophile in the aldol addition and the monitoring of the reaction time.

The oxidative decarboxylation with stoichiometric concentrations of H_2_O_2_ allows the synthesis of a carboxylic acid with a loss of a carbon atom and enables subsequent esterification, which reduces the polarity of products [28,35,38]. Since esterification often demands strong acidic or basic condition that potentially leads to a loss in the enantiopurity of the products, suitable conditions are required to maintain the *ee* in the product [38].

## Conclusion and outlook

The implementation of inherently sustainable enzyme-driven catalysts into chemical processes is advancing with the recent success in the development of novel biocatalytic carbon—carbon bond-forming reactions [10,11]. Recent efforts have expanded the notoriously small pool of nucleophiles of the so-called ‘pyruvic acid aldolase-mediated reactions. On the one hand, the finding of naturally occurring enzymes by high throughput methodologies allowed the tolerance of substituents at C3 of pyruvic acids. Hence, unprecedented stereoselectivity was identified by several novel enzymes, although, the examples with excellent diastereoselectivity are still limited to suitable nucleophile-electrophile pairing. On the other hand, protein engineering of the active site enabled aldolases and enzymes acting as aldolase to accept aliphatic and cyclic 2-oxo acid nucleophiles. This allows the synthesis of various precursors for bioactive compounds in a highly stereoselective fashion. Furthermore, the presented studies show class II aldolase-mediated reactions on a preparative scale with high substrate loading, which is advantageous for industrial applicability. The recent progress in this field offers an insight into the potential of these catalysts. It turns out that the obtained multifunctionalized building blocks are perfectly suited as starting point to provide a whole platform of different compounds as a diverse set of downstream routes are accessible and several one-pot protocols show their feasibility.

Biocatalytic aldol reaction still has limitations but is clearly on the upswing. One challenge is the supply of highly oxidized starting material for aldol reactions. Therefore, upstream reactions which provide the tedious 2-oxo acid and aldehyde substrates *in situ* would boost the applicability of pyruvic acid aldolase reactions [46]. It also would allow biomass-derived compounds as alternative starting material to construct the nucleophilic and electrophilic compounds that bear similar functionalization patterns.

## Abbreviations

HpcH: 4-hydroxy-2-oxo-heptane-1,7-dioic acid aldolase
GarL: 5-oxo-4-deoxy-d-glucarate aldolases
RhmA: 2-oxo-3-deoxy-l-rhamnonate aldolase
KphMT: oxopantoate hydroxymethyltransferase
